# Rare case of mixed epithelial and stromal tumor (MEST) of the kidney and its diagnostic and therapeutic approach: A case report

**DOI:** 10.1016/j.ijscr.2023.107882

**Published:** 2023-01-07

**Authors:** Wajiha Arshad, Asad Amir, Maria Naseer Malik, Shahzaib Maqbool, Muhammad Idrees Anwar, Ka Yiu Lee

**Affiliations:** a Department of Surgery Unit 2, Holy Family Hospital, Rawalpindi, Pakistan; b Department of surgery Holy Family Hospital Rawalpindi, Pakistan; c Department of Health Sciences, Mid Sweden University, Östersund, Sweden

**Keywords:** Mixed epithelial stromal tumor (MEST), Radical nephrectomy, Cystic lesion

## Abstract

**Introduction and importance:**

Mix epithelial and stromal tumor (MEST) is a benign biphasic renal lesion composed of solid as well as cystic components lining tubular and cystic spaces of kidney. There are very few cases of such variety have been reported with perspective to renal involvement. Herein we have reported a rare case of MEST involving left renal tissue and sparing surrounding tissues.

**Case presentation:**

A 20 years old female presented to surgical outpatient department with complaint of amenorrhea and left flank pain as well as heaviness for 1 year. Patient was vitally stable and cooperative. On physical examination left flank mass was palpated and ultrasound and CT scan imaging was also showing left renal mass confined to upper, middle and lower portion of the kidney while renal capsule, adrenal gland and ureter were spared. On histological examination showed multi-cystic structures with variably sized simple cysts lined by hobnailed epithelium with clear cells. Septa show ovarian type fibrous stroma with variable inflammation and immature nephrogenic elements. A final diagnosis of MEST was made. Therefore, radical nephrectomy with trans-peritoneal approach was done.

**Clinical discussion:**

MEST is a benign tumor of renal tissue that is confined to the renal parenchyma rather than involvement of surrounding structures as occurred in our case. Due to benign nature of the disease involvement of renal capsule and adrenal gland is less likely. The choice of treatment is radical nephrectomy through transperitoneal approach.

**Conclusion:**

MEST is a rare diagnosis thought case now start reporting since last decade, however, it’s still a rare entity to be reported. USG and CT scan are investigating modalities along with histopathological correlation to reach the diagnosis.

## Highlights

• Mixed epithelial and stromal tumor (MEST) of kidney is a benign lesion of kidney that correspond to a rare diagnosis.
• Ultrasound and CT scan are investigating modalities for diagnosis of MEST.
• Radical nephrectomy through transperitoneal approach is treatment of choice for MEST of kidney.

## 1 Introduction

Mixed epithelial and stromal tumor of the kidney (MEST) is a rare and typically benign tumor, common in perimenopausal women [1], [2]. About hundred cases have been reported and it was first reported by Micheal and Syucek in 1998 [3]. Mix epithelial and stromal tumor (MEST) is a benign biphasic renal lesion composed of solid as well as cystic components lining tubular and cystic spaces of kidney [2]. There are very few cases of such variety have been reported with perspective to renal involvement. Herein we have reported a rare case of MEST involving left renal tissue and sparing surrounding tissues. The SCARE guidelines were followed in writing of this case report [4].

## 2 Case presentation

**Fig. 1:**
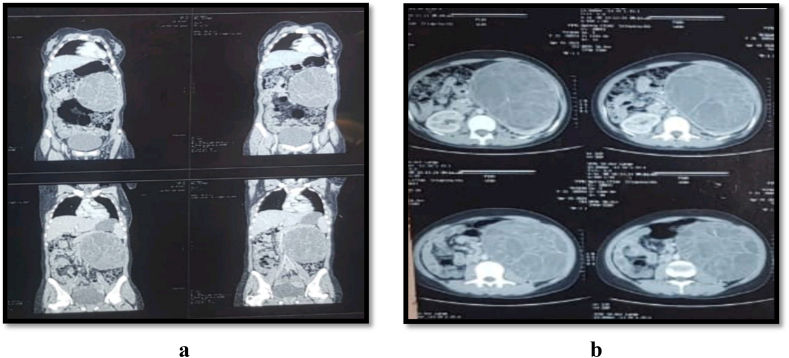
a and b Showing the CT scan imaging of tumor mass involving left renal tissue in different planes.

A 20-year-old female presented with a complaints of amenorrhea, left flank pain and heaviness from 1 year. There was no associated weight loss, and patient was vitally stable. Flank pain was gradual in onset and progress and diffuse in nature. On physical examination patient was of normal height and weight but systemic examination a left renal mass was palpated that felt homogeneous in nature. At presentation Patient was vitally stable and laboratory investigations ordered were also with in normal standard reference ranges expect Hb level, that was slightly towards lower normal limit. On ultrasound study a left renal mass involving the major portion of left renal tissue with homogeneous density was observed. The ultrasound also revealed a tumor like image that was occupying the whole left kidney (upper, middle, and lower borders). On further investigations computerized tomography (CT) scan revealed size of 180 mm with no invasions to renal capsule, renal vessels, or ureters or homolateral adrenal gland as shown in Fig. 1a and b. Infiltrative borders of tumor mass suggested as an untypically renal cell carcinoma. Therefore, radical nephrectomy was done.

**Fig. 2:**
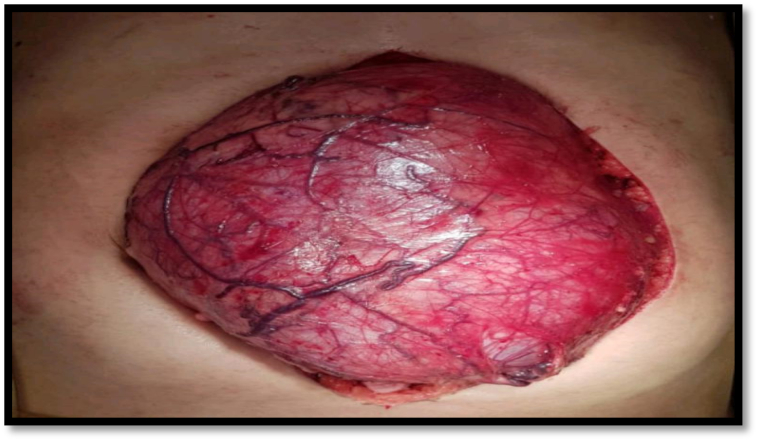
Showing Gross intraoperative presentation of tumor and its size in different dimensions.

During operation the renal capsule was found intact, and the renal artery was clamped, no complications were faced during operation and patient had quick and perfect recovery post operatively. Gross examination of the specimen revealed a tumor dimension of 180 × 150 × 110 mm involving the whole kidney middle upper and lower lobe as shown in Fig. 2. Ureters, renal vein, renal capsule, perinephric mass uninvolved by tumor. However renal sinus fat was completely replaced by tumor. Cut surface of the tumor shows multiple small cysts, filled with clear fluid.

**Fig. 3:**
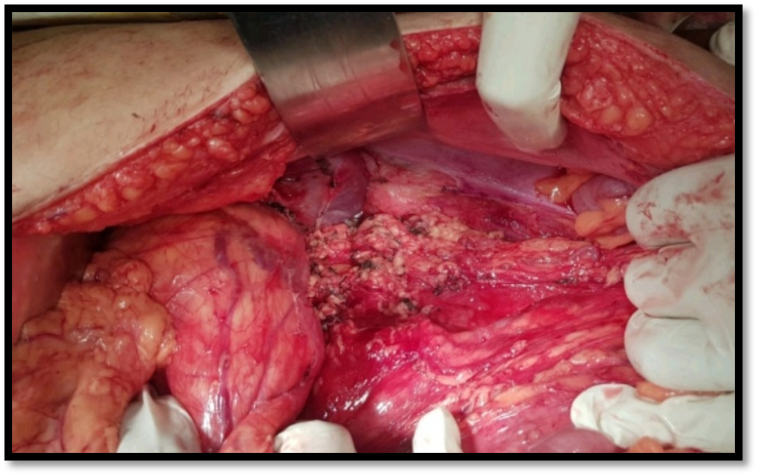
Showing transperitoneal approach of radical nephrectomy for removal of tumor mass.

Patient was informed about the nature of the disease and patient was prepared for radical nephrectomy after getting anaesthesia fitness. Tumor mass was removed by radical nephrectomy that was performed through transperitoneal approach as shown in Fig. 3 and the resected specimen was preserved for microscopic and histological analysis. Microscopic and histological sections showed multi-cystic structures with variably sized simple cysts lined by hobnailed epithelium with clear cells. Septa show ovarian type fibrous stroma with variable inflammation and immature nephrogenic elements. Final histopathological diagnosis was Mixed Epithelial and Stromal Tumor (MEST) and patient was placed on strict follow up, irrespective of that the malignant transformation and recurrence history is very rare of natural history of MEST.

## 3 Discussion

Mixed epithelial and stromal tumors of the kidney are recently classified as distinct neoplasm, Imaging studies reveal solid and cystic lesions and are diagnostic for it. MEST is prevalent from 19 years to 78 years of age with distinct female predominance (male to female ratio 1:6) [2]. Etiology of MEST is unknown though it is suggested to be caused due to hormonal pathogenetic mechanisms. Rare cases of men and paediatrics patients were found in overall reported cases of MEST in literature [5], [6] mean size of MEST was reported in 8 case series of 2.9 cm ranging 0.5 cm to 10 cm [5], [6]. Changing hormonal levels in females or males mostly due to hormonal replacement therapy commonly estrogen replacement is suggestive of the main cause of MEST.

Changing levels of hormones causes proliferation of ectopic or fetal mesenchymal cells located in the kidney, with the ability to differentiate into epithelial and stromal cells [7]. However, in our cases like some cases reported earlier we didn't have any history of hormonal replacement therapy and no evidence of hormonal receptor expression was found either [8]. Therefore, we suggest not all cases of MEST implicate hormonal mechanisms. Clinically patients are usually present with flank pain or palpable mass and mostly suspected as upper urinary tract infection. [3] mostly its unilateral, while bilateral and multiple cases are very rarely reported [9].

Tumor has been classically classified into solid and cystic components. In some cases, solid part is predominant while in other cystic components it is predominant [10], [11] Microscopically the tumor is biphasic comprising mesenchymal and epithelial components [12]. In our case it was found on CT that a tumor mass covering the whole kidney and no adjacent invasions. This was one of reasons why we thought it was an atypical MEST. Microscopically tumor is biphasic comprising of mesenchymal and epithelial components [12] Mesenchymal elements is characterized by fascicles of spindle cells with variable degree of smooth muscle, fibroblast, or myofibroblast differentiation while epithelial element is an integral part of the neoplasm that vary from regular tubules to more complex tubulo-papillary structures with or without cystic dilations [7], [11] In our case multi-cystic structures with variably sized simple cysts lined by hobnailed epithelium with clear cells. The septae show ovarian type fibrous stroma with variable inflammation and lack immature nephrogenic elements.

Differentiation of MEST from other renal neoplasms clinically and pathologically. Major differential diagnosis includes:

• Cystic nephroma (CN) CN owns considerable overlap with MEST in clinical behaviour as well as in morphologic attributes as both consists of epithelial and stromal components [13]. In comparison to MEST, CN own larger cysts, thinner septa, and lower prevalence of stromal to epithelial ratio [2], [7]
• Primary renal synovial sarcoma. It is characterized by gross or microscopic cysts and tubules lined by hobnail epithelium with immunoreactivity for cytokeratin in epithelium element, which is found in malign-ant MEST as well [14], [15] in our case hobnail epithelium was found with septae that shows ovarian type stroma which differentiate it for MEST as ovarian stroma is not found in primary renal synovial sarcoma.
• Congenital mesoblastic nephroma. It consists of a solid mass composed of spindle stromal cell elements with entrapped renal tubules and always involve renal parenchyma. [11]

Multi-cystic renal cell carcinoma. Lack of aggregates of clear cells is a differentiating characteristic of MEST from multi-cystic renal cell carcinoma. MEST have generally benign behaviour in most cases, but some aggressive MEST have been reported in literature so far [5], [16], [17]. Due to the rarity of MEST, whether true malignant transformation is still not known.

## 4 Conclusion

In conclusion, Mixed Epithelial and Stromal tumor of the kidney is predominantly benign type, but it can exist in metastatic form as well. While managing cases of cystic tumors in middle aged, perimenopausal women or men with the history of hormonal therapy, the possibility of MEST should be considered. Surgical intervention (partial or radical nephrectomy) is preferred.

## Patient's perspective and follow up

The patient was satisfied with given treatment and she was also encouraged to follow up for that particular case in order to assess any unwanted complications and disease spread though it was not evident on CT scan findings, but still follow up was encouraged for better understanding of disease course.

## Consent

Written informed consent was obtained from the patient for the publication of this case report and accompanying images. A copy of the written consent is available for review by the Editor-in-Chief of this journal on request.

## Source of funding

N/A.

## Ethical approval

Ethical approval has been exempted by our institution because this is a case report and no new technique were carried out.

## Registration of research studies

N/A.

## Provenance and peer review

Not commissioned, externally peer-reviewed.

## CRediT authorship contribution statement

Wajiha Arshad: Writing-Original draft preparation & editing.

Asad Amir, Maria Naseer Malik: Writing-review & editing.

Muhammad Idrees Anwar: Performed surgery & writing-review.

Shahzaib Maqbool, Ka Yiu Lee: Writing-review & editing.

All authors read and approved the final manuscript.

## Declaration of competing interest

N/A.
